# Study protocol for a randomized controlled trial on the effect of the Diabetic Foot Guidance System (SOPeD) for the prevention and treatment of foot musculoskeletal dysfunctions in people with diabetic neuropathy: the FOotCAre (FOCA) trial I

**DOI:** 10.1186/s13063-019-4017-9

**Published:** 2020-01-13

**Authors:** J. S. S. P. Ferreira, R. H. Cruvinel Junior, E. Q. Silva, J. L. Veríssimo, R. L. Monteiro, D. S. Pereira, E. Y. Suda, C. D. Sartor, I. C. N. Sacco

**Affiliations:** 1 Department of Physical Therapy, Speech, and Occupational Therapy, School of Medicine, University of São Paulo, Rua Cipotânea, 51 - Cidade Universitária, São Paulo, São Paulo 05360-160 Brazil; 2 Department of Physical Therapy, Federal University of Amapá, Amapá, Brazil; 3 Department of Physical Therapy, Ibirapuera University, São Paulo, SP Brazil

**Keywords:** Diabetic foot, Preventive care, Foot-related exercises, Self-management, eHealth, Musculoskeletal function, Rehabilitation technology

## Abstract

**Background:**

This study is part of a series of two clinical trials. Taking into account the various musculoskeletal alterations of the foot and ankle in people with diabetic peripheral neuropathy (DPN) and the need for self-care to avoid more serious dysfunctions and complications, a self-manageable exercise protocol that focuses on strengthening the foot muscles is presented as a potentially effective preventive method for foot and gait complications. The aim of this trial is to investigate the effect of a customized rehabilitation technology, the Diabetic Foot Guidance System (SOPeD), on DPN status, functional outcomes and gait biomechanics in people with DPN.

**Methods/design:**

Footcare (FOCA) trial I is a randomized, controlled and parallel two-arm trial with blind assessment. A total of 62 patients with DPN will be allocated into either a control group (recommended foot care by international consensus with no foot exercises) or an intervention group (who will perform exercises through SOPeD at home three times a week for 12 weeks). The exercise program will be customized throughout its course by a perceived effort scale reported by the participant after completion of each exercise. The participants will be assessed at three different times (baseline, completion at 12 weeks, and follow-up at 24 weeks) for all outcomes. The primary outcomes will be DPN symptoms and severity classification. The secondary outcomes will be foot–ankle kinematics and kinetic and plantar pressure distribution during gait, tactile and vibration sensitivities, foot health and functionality, foot strength, and functional balance.

**Discussion:**

As there is no evidence about the efficacy of rehabilitation technology in reducing DPN symptoms and severity or improving biomechanical, clinical, and functional outcomes for people with DPN, this research can contribute substantially to clarifying the therapeutic merits of software interventions. We hope that the use of our application for people with DPN complications will reduce or attenuate the deficits caused by DPN. This rehabilitation technology is freely available, and we intend to introduce it into the public health system in Brazil after demonstrating its effectiveness.

**Trial registration:**

ClinicalTrials.gov, NCT04011267. Registered on 8 July 2019.

## Background

Diabetic peripheral neuropathy (DPN) is one of the most prevalent chronic complications of diabetes mellitus (DM) [1]. It is estimated that between 12 and 50% of people with DM have some degree of DPN [2]. As a result of the sensorimotor impairments caused by DPN, several musculoskeletal dysfunctions are present. These include alterations in the joints surrounding tissues, reducing their range of motion [3–5], and reduction in functionality and strength due to deterioration of the intrinsic foot and lower leg muscles [6–8].

People with DM commonly present with alterations in gait biomechanics, such as reduced gait speed, decreased ankle dorsi and plantarflexion [5, 9], delayed muscle activations in the leg and thigh [5, 10–15], reduced ankle and increased hip joint moments [16–18], and altered plantar pressure distribution patterns that increase the risk of developing plantar ulcers [19–23]. Due to these changes, individuals with DM may have difficulties performing their daily locomotor activities, which compromises their functionality and negatively impacts their quality of life [24–26].

Recent studies have evaluated the effects of performing specific exercises for musculoskeletal deficits in people with DPN on clinical and biomechanical variables and have concluded that they can reduce peak plantar pressure during gait [27–33], minimize DPN symptoms and loss of sensitivity [30, 33], increase the mobility of the foot–ankle complex and the first metatarsophalangeal joint [27, 32–34], and maximize the strength of the foot–ankle muscles [30, 34, 35]. However, for some of the above-cited interventions successful results were not maintained at follow-up [36], which suggests that self-care after any intervention is pivotal for the maintenance of the health benefits achieved when managing DM and its chronic complications. To accomplish this goal, an intervention program should include various care modalities—such as education, self-assessment, foot self-management, foot self-care, medication, and proper nutrition—in addition to the most current approaches, as exercise can help to prevent musculoskeletal complications due to disease progression [37].

Education through software or booklets that provide guidelines for self-care and at-home exercises can be potentially successful alternatives to traditional treatments [38–40]. The accessibility of and advances in modern technology have made it possible to use electronic devices to improve adherence to health prevention and treatment, especially for chronic diseases [41].

Ferreira et al. [40] have developed and validated free, publicly available software, the Diabetic Foot Guidance System (SOPeD) (www.soped.com.br), with general guidelines on foot self-care and self-assessment and a personalized protocol including balance, strengthening, and stretching exercises for the treatment and prevention of the most common foot–ankle musculoskeletal deficits due to DM and DPN. This software is designed to provide and monitor exercises, facilitate self-care, and improve autonomous performance of daily living tasks [40]. However, its efficacy for the treatment of people with DM and DPN has not yet been proven and, to date, no studies have been performed to evaluate the effects of a rehabilitation technology capable of personalizing specific therapeutic foot exercises aimed at prevention and treatment of musculoskeletal deficits and complications in people with DPN.

The primary aim of this superiority randomized controlled, singe-blind, two-parallel-arm trial, the Footcare (FOCA) trial I, is to investigate the effects of the customized foot-related exercise software SOPeD on DPN symptoms and severity after 12 weeks (intervention completion) and 24 weeks (follow-up) in patients with DPN. The secondary aims are to investigate the effects of this intervention at 12 and 24 weeks on foot–ankle biomechanics and plantar pressure during gait, tactile and vibration sensitivities, foot health and functionality, foot strength, and functional balance.

We hypothesize that the therapeutic customized exercise protocol will reduce DPN symptoms and severity as well as improve foot health and functionality status, plantar sensitivity (tactile and vibration perceptions), and functional balance scores. We also hypothesize that the protocol will produce beneficial biomechanical changes during gait that denote an improvement in foot–ankle kinematics and in the plantar pressure distribution. Such changes may include: 1) an increase in the ankle range of motion in the sagittal plane; 2) an increase in the ankle extensor moment and power; 3) an increase in the ankle flexor moment and eccentric power during the heel-strike phase; 4) a reduction in peak pressure over risky areas; and 5) improved foot rollover by redistributing plantar pressure during gait.

It is important to highlight that intervention programs in this population rarely include any foot-related exercises focusing on the most common and frequently described musculoskeletal foot–ankle deficits. The present proposal uses a new paradigm that focuses on autonomous and independent use of a rehabilitation technology to improve self-care and management, with the aim of enhancing compliance to preventive strategies in people with DM.

## Methods and design

### Trial design

This study is part of a series of two clinical trials: the FOCA trial I (SOPeD intervention) and FOCA trial II (booklet intervention). The FOCA trial I is a superiority, single-blind, randomized controlled trial with two parallel arms where participants will be randomized into one of two groups: the intervention group that will perform foot-related exercises included in the SOPeD; and the control group that will not receive any specific intervention besides the treatment recommended by a health care professional. This trial will have an allocation ratio of 1:1.

People with DM and DPN will be recruited from the Department of Endocrinology of the Hospital das Clínicas of the School of Medicine of the University of São Paulo, Brazil, and will be referred to a physical therapist who will perform the group allocation. The participants will then be referred to another physical therapist who will perform the initial blind assessment. All participants allocated to the intervention group will participate in a protocol of monitored and customized therapeutic exercises for the foot–ankle complex using the SOPeD software three times a week for 12 weeks [40]. After the 12-week period, the intervention group participants will be encouraged to continue exercising until the end of the study following the same schedule set during the intervention period. The participants allocated to the control group will not receive any specific intervention besides the regular treatment recommended by health care professionals (doctors, nurses, and podiatrists). If proven effective, the benefits of the foot-related exercise protocol will be explained and offered to all control participants at the end of the study.

All participants will be assessed at baseline, 12 weeks (end of intervention) and 24 weeks (follow-up). Assessments will record DPN symptoms and severity (primary outcomes) and all other secondary outcomes.

The Consolidated Standards of Reporting Trials (CONSORT) 2010 guidelines will be followed. The study was approved by a research ethics committee (CAAE: 90331718.4.0000.0065) and was registered at ClinicalTrials.gov on 8 July 2019 (study identifier NCT04011267).

### Study setting

The assessments will be performed at the Laboratory of Biomechanics of Human Movement and Posture at the Physical Therapy, Speech and Occupational Therapy Department of the School of Medicine of the University of São Paulo, São Paulo, Brazil. The participants allocated to the intervention group will perform the exercises by themselves in their homes, but the first session will be in an ambulatory setting to provide them with a reliable therapeutic environment and treatment assistance. This first session will be conducted by a physical therapist who will teach and supervise the correct execution of the exercises performed while using the software.

### Participants and recruitment

This study is currently recruiting patients (study start date 1 August 2019) with a medical diagnosis of DM and DPN from the Department of Endocrinology of the Hospital das Clínicas of the School of Medicine of the University of São Paulo through telephone contact. Sixty-two patients with DPN will be recruited. The potential participants will be interviewed by telephone and, upon selection, assessed in the laboratory to confirm all eligibility criteria. This first laboratory assessment will represent the baseline condition (blind assessment).

### Eligibility criteria

#### Inclusion criteria

Adults (age 18–65 years) with DM type 1 or 2 and mild, moderate, or severe DPN confirmed using fuzzy software (www.usp.br/labimph/fuzzy; score ≥2) who have the ability to walk independently in the laboratory and have access to an electronic device (computer, mobile device, tablet, etc.) and the SOPeD exercise software will be included in the study.

#### Exclusion criteria

Patients with any of the following exclusion criteria will be excluded from the study: hallux amputation or total amputation of the foot; a history of surgical procedures in the knee, ankle, or hip; a history of arthroplasty and/or indication of lower limb arthroplasty throughout the intervention period; a diagnosis of neurological and/or rheumatologic diseases; wearing an off-loading device; major vascular complications; severe retinopathy; ulceration not healed for at least 6 months and/or active lower limb ulcers. Additionally, patients who use lower limb orthosis during the intervention period, are unable to provide consistent information, undergo a physiotherapy intervention throughout the intervention period, or report a score between 12 and 21 (probable depression) on the Hospital Anxiety and Depression Scale will also be excluded from the study.

### Procedure

**Fig. 1:**
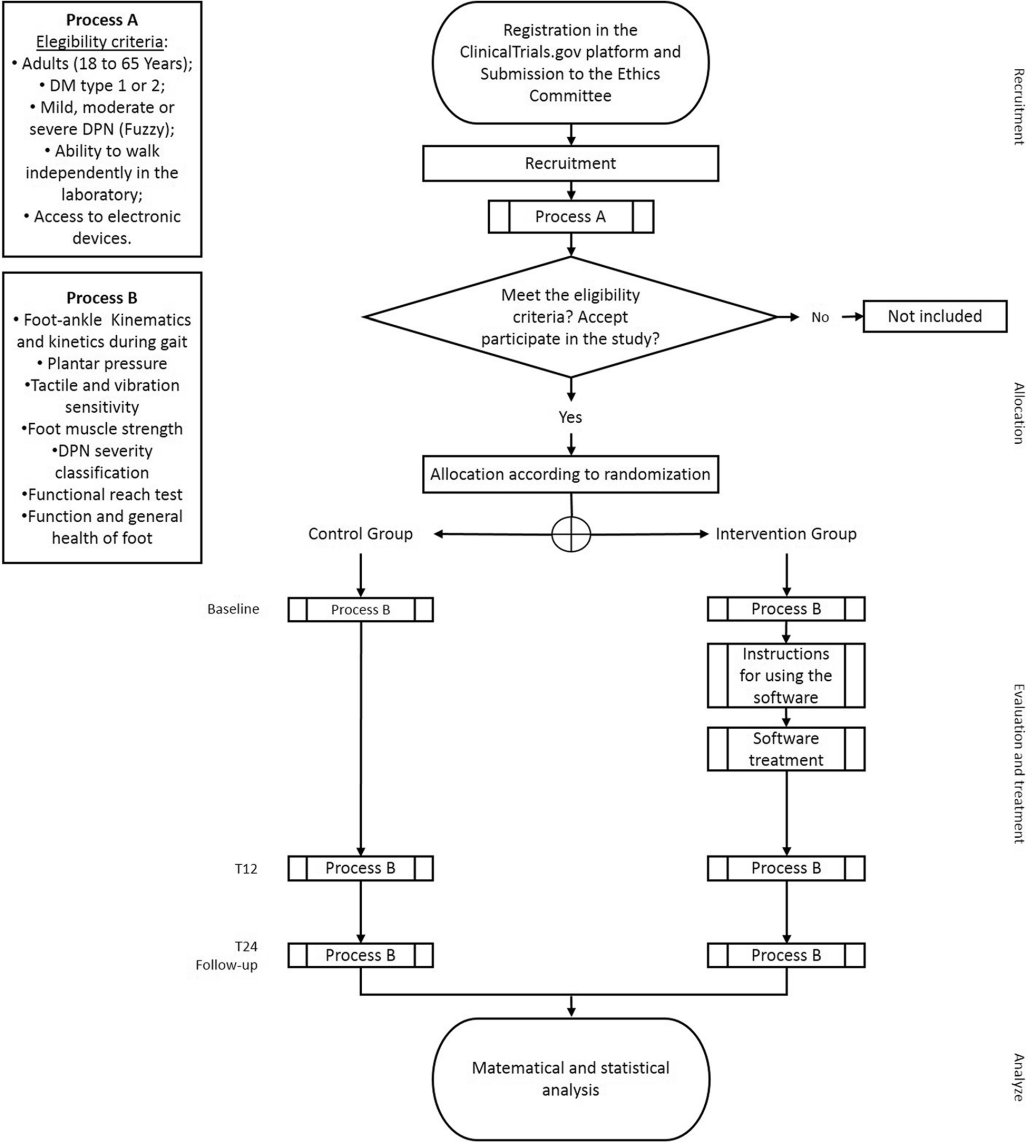
Consolidated Standards of Reporting Trials (CONSORT) flow diagram flow chart illustrating the process of the Footcare (FOCA) trial I. DM diabetes mellitus, DPN diabetic peripheral neuropathy

The trial protocol will follow all recommendations established by the Standard Protocol Items Recommended for Clinical Trials (SPIRIT) 2013 statement [42] (the checklist can be found in Additional file 1). Figure 1 presents the design and flowchart of the protocol according to the CONSORT 2010 standards [43].

### Randomization, allocation, and blinding

pt?>The randomization schedule has been prepared using Clinstat software [44] by an independent researcher (Researcher 1) who is not aware of the numeric coding for the control group and intervention group. A numeric block randomization sequence is kept in opaque envelopes.

Potential participants will be assessed through an initial screening that consists of checking the eligibility criteria, classifying the DPN severity, and identifying those with a lower probability of adherence to the intervention due to depression. After the initial screening, participants who meet the inclusion criteria will be randomized to either the intervention group or control group. After the agreement to participate and assignment in the research, the allocation of the groups will be made by another independent researcher (Researcher 2) who is also unaware of the codes. All participant personal data will be kept confidential before, during, and after the study by encoding the participants’ names. Only the physiotherapist (Researcher 3), who is the main researcher responsible for the intervention, is aware of who is receiving the intervention. Researcher 3 is also responsible for the remote monitoring of the intervention by the SOPeD software and by telephone. One physiotherapist and an occupational therapist (Researchers 4 and 5), who are also blind to the treatment allocation, will be responsible for all clinical, functional and biomechanical assessments. Researchers 3, 4, and 5 are blind to the block size used in the randomization procedure. The trial statistician will also be blind to treatment allocation until the main treatment analysis has been completed. The trial design is open label where only the outcome assessors are blinded so unblinding will not occur.

### Trials arms

#### Control group

Participants in the control group will not receive any specific intervention besides the treatment recommended by the health care team (doctors, nurses, and podiatrists), which will include pharmacological treatment as well as self-care and foot care recommendations as per international consensus. If proven effective, the benefits of the foot-related exercises protocol will be explained and offered to all control group participants at the end of the study.

#### Intervention group

Patients in the intervention group will perform customized foot-related exercises included in the SOPeD software three times per week at home for a 12-week period. These will be remotely supervised by Researcher 3 after the first in-person supervised session at the department. They will receive access and all instructions on how to use the tool on the first day. During the follow-up period, intervention group participants will be encouraged to follow the same schedule set by the project till the end of the study (24 weeks after allocation), but will not be remotely monitored, and will be encouraged to continue exercising in the future.

### Outcomes and measures

#### Participant timeline

Participants in the intervention group and control group will be assessed at the preintervention baseline (T0), at the end of the 12-week intervention (T12), and at follow-up 24 weeks after baseline (T24). A physiotherapist and an occupational therapist (Researchers 4 and 5) who are blinded to group allocation will perform all the assessments. All participants will maintain contact with Researcher 3 throughout the follow-up period through the web software, email, and telephone. Table 1 shows the schedule of enrollment, interventions, and assessment according to the SPIRIT guidelines [42].

**Table 1:**
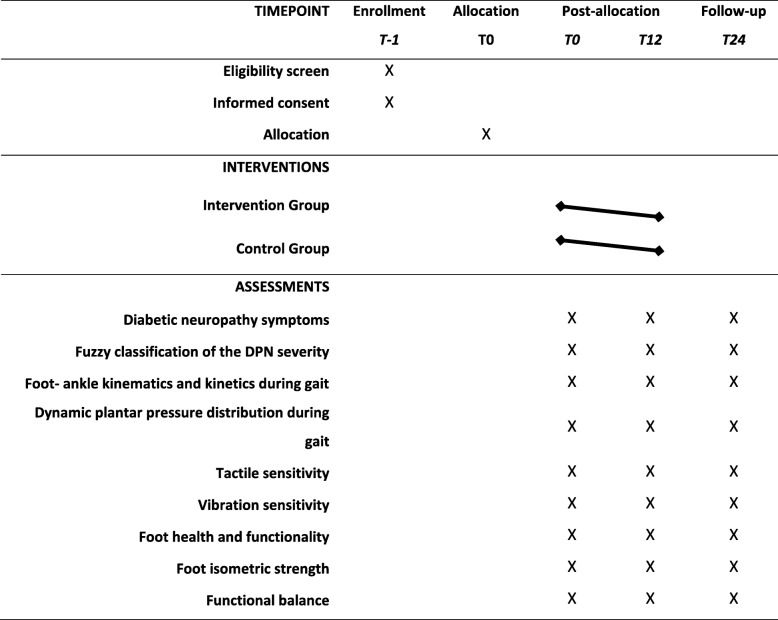
Schedule of enrollment, interventions and assessment of the Footcare (FOCA) trial I, following the Standard Protocol Items Recommended for Clinical Trials (SPIRIT) guidelines

#### Screening measures

An initial anamnesis will be performed to check the eligibility criteria, including clinical, anthropometric, and demographic characteristics of all participants. The classification of DPN severity will be determined using the Decision Support System for Classification of Diabetic Polyneuropathy [45, 46] (www.usp.br/labimph/fuzzy). Participants with scores ≥2.0 (mild DPN) will be included in the study. Those who score between 12 and 21 (probable depression) on the Portuguese version of the Hospital Anxiety and Depression Scale will not be included [47].

### Measures of primary and secondary outcomes

The quantification of DPN symptoms and classification of DPN severity will be the primary outcomes. Foot–ankle kinematics and kinetics during gait, plantar pressure distribution during gait, tactile and vibration sensitivities, foot health and functionality, foot strength, and functional balance constitute the secondary outcomes.

#### DPN symptoms

DPN symptoms will be assessed by the Brazilian version of the Michigan Neuropathy Screening Instrument [48]. This self-administered questionnaire is comprised of 15 questions about symptoms and events related to foot sensitivity. The answers are summed to get a total score ranging from 0 to 13 (with 13 representing the worst DPN).

#### Fuzzy classification of DPN severity

The decision support system for the classification of DPN is based on fuzzy logic using three domains: signs and symptoms extracted from the Michigan Neuropathy Screening Instrument, tactile sensitivity through the number of nonsensitive areas using a 10-g monofilament, and vibration sensitivity evaluated by a tuning fork (128 Hz) and classified as absent, present or diminished. The software produces a score from 0 to 10, with a higher score indicating more severe DPN.

#### Tactile sensitivity

Tactile sensorial deficits will be evaluated by a 10-g monofilament [49] in four plantar areas (plantar face of the hallux and first, third, and fifth metatarsal heads). The areas will be evaluated in a random order, and the participant will not be allowed to view the monofilament. The number of areas where the participant does not feel pressure will be evaluated [49]. The greater the number of areas marked, the greater the impairment of tactile sensitivity.

#### Vibration sensitivity

Vibration sensitivity will be assessed by a tuning fork (128 Hz) perpendicular to the dorsal region of the distal hallux phalanx at constant pressure. The participant will report the moment they no longer feel the vibration of the tuning fork, and the evaluator must time the interval between which the participant reports that they cease to feel the vibration and the moment the evaluator ceases to feel the vibration in their hand [50]. Values smaller than 10 s will be classified as normal vibratory sensitivity; values greater than 10 s will be classified as decreased vibratory sensitivity; if the participant does not perceive the vibration imposed by the tuning fork, it will be classified as absent vibratory sensitivity [50].

#### Foot health and functionality

The Brazilian version of the Foot-Health Status Questionnaire (FHSQ-BR) [51] will be used to determine foot health. This instrument is divided into three domains, and this study will use domains I and II. Domain I evaluates the foot in four dimensions: pain, function, footwear, and general health. Domain II evaluates the general state of health in four dimensions: general health, physical activity, social capacity, and vitality. Domains I and II are comprised of questions with answers in affirmative sentences and a Likert-type scale. Domain III is comprised of the collection of participants’ general demographic data. Domains I and II receives a score from 0 to 100, where 100 expresses the best condition and 0 the worst. The data will be analyzed using the FHSQ version 1.03 software (Care Quest—Researching Healthcare Solutions, Australia).

**Fig. 2:**
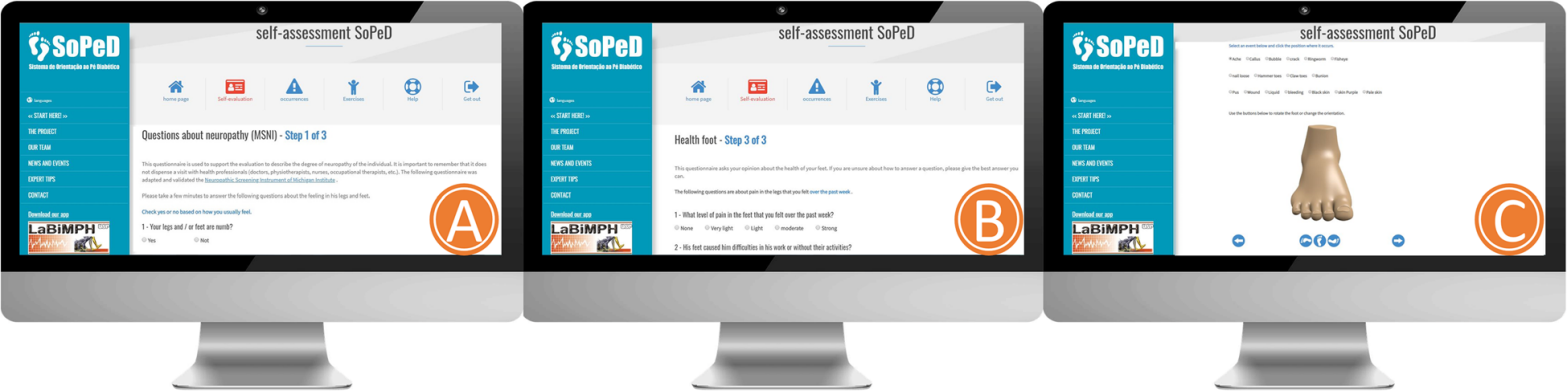
Layout of the Diabetic Foot Guidance System (SOPeD). (**a**) Self-assessment of common foot problems with diabetes mellitus and diabetic peripheral neuropathy. (**b**) Report of the assessment. (**c**) The foot physical examination

In addition to the face-to-face assessment, every 30 days the participants will self-assess their DPN symptoms as well as the health status of their feet by completing both the FHSQ-BR questionnaire and undergoing a physical examination of the foot using the SOPeD software (www.soped.com.br), in which the participant can drag markers that represent major foot changes (calluses, cracks, fissures, mycoses, toe deformities, ulcers, and amputations) onto images of both feet (Fig. 2). At the end of these monthly evaluations, the participant and the main researcher will be provided with a report related to the patient’s conditions and whether or not they are still able to perform the exercise protocol.

#### Foot isometric strength

The muscle isometric strength of the flexor muscles of the hallux and lesser toes will be evaluated using the emed-q100 pressure platform (novel, Munich, Germany) according to a test protocol previously described by Mickle et al. [52]. The participant will stand with the evaluated foot centered on the pressure platform and will be instructed to push down as hard as possible using only the hallux and toes, particularly on the metatarsophalangeal joints and not the hallux interphalangeal joint. A physiotherapist will determine whether the participant lifted the heel and will inspect fluctuations in the line of gravity and trunk posture during each trial. If any changes are observed in the line of gravity or positioning of the heel or trunk, the trial will be excluded. Three valid trials will be completed on each foot (left and right), and the plantar regions corresponding to the hallux and toes will be identified. To determine such areas, novel-win Multimask software v. 9.35 (novel, Munich, Germany) will be used. Maximum force (N) will be normalized by body weight and analyzed for the hallux and toe areas separately as well as for the whole foot.

#### Functional balance

Functional balance will be evaluated using the Functional Reach Test proposed by Duncan et al. [53]. The patient will stand barefoot with the foot dorsal region perpendicular to the wall, with both feet parallel in a comfortable position, without touching the wall, and with the shoulder flexed at 90° and the elbow extended. The hands will be closed. A tape measure will be attached to the wall parallel to the floor and positioned at the height of the patient’s acromion. The initial measurement will correspond to the position in which the third metacarpal touches the tape. The participant will then be instructed to lean forward as much as possible without losing balance, flexing the hips, or taking a step. Displacement will be checked on the tape measure. Three trials will be performed, and the mean of the trials will be used for statistical purposes. The greater the distance achieved, the better the functional balance.

#### Foot–ankle kinematics and kinetics during gait

The foot–ankle kinematic parameters during gait will be acquired by eight infrared cameras at 100 Hz (Vicon® VERO, Vicon Motion System Ltd., Oxford Metrics, Oxford, UK). Ground reaction forces will be acquired by a force plate (AMTI OR-6-1000, Watertown, MA, USA) with a sampling frequency of 100 Hz embedded in the center of a 10-m walkway. Force and kinematic data acquisition will be synchronized and sampled by a 16-bit analog-to-digital converter.

The laboratory coordinate system will be established at one corner of the force plate, and all initial calculations will be based on this coordinate system. Each lower-limb segment (shank and thigh) will be modeled based on surface markers as a rigid body with a local coordinate system that coincides with the anatomical axes. Translations and rotations of each segment will be reported relative to the neutral positions defined during the initial static standing trial. All joints will be considered spherical (i.e., with three rotational degrees of freedom). Forty-two reflexive-passive markers (diameter = 9.5 mm) will be positioned on both lower limbs following the Plug-In Gait setup protocol and the Oxford Foot Model (OFM) setup protocol [54]. The foot will be modeled by the OFM, which has been tested for reproducibility regarding its applicability to foot biomechanics analysis during gait [55, 56].

After the volume calibration and the static trial acquisition, participants will be advised to walk at their comfortable self-selected speed along a 10-m track, with a maximum variation of 5% between measurements, thus ensuring that the same speed is maintained in all assessments (T0, T12, T24) of the same participant. Two photoelectric cells (CEFISE, Model Speed Test Fit, Nova Odessa, Brazil) 6 m apart will be used to verify the self-selected speed and to check the trials and assessments. After complete habituation to the laboratory environment, five valid steps will be acquired on each side during gait.

The automatic digitizing process, three-dimensional reconstruction of the markers’ positions, and filtering of kinematic data will be performed using motion capture Nexus software (NEXUS 2.6, Vicon Motion System Ltd., Oxford Metrics). Kinematic data will be processed using a zero-lag second-order low-pass filter with cutoff frequency of 6 Hz. Ground reaction force data during walking will be processed using a zero-lag low-pass Butterworth fourth-order filter with cutoff frequency of 50 Hz.

The bottom-up inverse dynamics method will be used to calculate the net moments in the sagittal of the ankle considering the inertial properties of the segments [57]. For the calculation of the ankle power, the calculated joint moment and the angular velocity of the ankle in the sagittal plane will be considered. The temporal series of the joint angles and moments will be calculated using the Plug-In Gait and OFM in the Vicon Nexus software. Calculation of all discrete variables from the time series will be performed using a custom written MATLAB function (MathWorks, Natick, MA, USA).

The following ankle kinematic variables will be analyzed: 1) maximum dorsiflexion during the heel-strike phase; 2) maximum plantarflexion during the push-off phase; 3) maximum dorsiflexion during the toe-off phase; and 4) dorsiflexion range of motion in the sagittal plane during the stance phase. The foot kinematic variables that will be analyzed include: 1) hindfoot to forefoot rotation; 2) transverse plane angle between the first and second metatarsal bones; 3) transverse plane angle between the second and fifth metatarsal bones; 4) maximum inversion of the calcaneus (frontal plane); 5) maximum eversion of the calcaneus (frontal plane); and 6) deformation of the medial longitudinal arch. The ankle kinetic variables that will be analyzed are the ankle flexor moment peak normalized by body weight times height during the heel-strike phase and the ankle extensor moment peak at approximately 80% of the gait support phase, corresponding to the propulsion phase.

#### Plantar pressure distribution during gait

A pressure platform (emed-q100, novel) will be used to evaluate the pressure distribution during walking. The pressure platform is 700 × 403 × 15.5 mm with 6080 sensors and a resolution of four sensors per square centimeter when data is collected at 100 Hz. Participants will be invited to walk barefoot across the platform at a comfortable self-selected speed (the same as in the kinematic trials), and data for both feet will be collected. For the analysis of the regions of interest, an anatomical mask that divides the foot into seven anatomical plantar regions—heel, midfoot, medial forefoot, medium forefoot, lateral forefoot, hallux, and two to five toes—will be analyzed. The anatomical mask will be performed from the integration of the plantar pressure and kinematic gait of the multisegmented OFM. Peak pressure (kPa) and pressure–time integral (kPa*s) will be analyzed for each region of interest in both feet [58].

#### Intervention

Participants allocated to the intervention group will receive a customized foot–ankle therapeutic exercise protocol for strengthening and improving functionality based on the SOPeD software.

The exercise protocol (eight exercises per session) will be performed three times a week for 12 weeks and remotely supervised by the physiotherapist (Researcher 3). Each session will last for 20 to 30 min. The participants in the intervention group will be encouraged to continue exercising using the same regimen established in the 12-week intervention during the follow-up period (12 additional weeks).

**Fig. 3:**
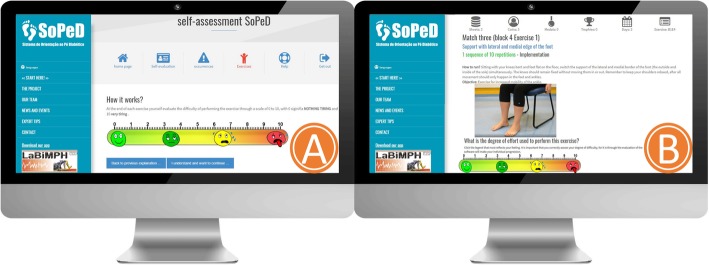
Diabetic Foot Guidance System (SOPeD). (**a**) perceived effort scale to be filled after each exercise completion. (**b**) Layout of the exercises page with a video, audio and written instructions for each exercise

The therapeutic exercise protocol and SOPeD have been described in detail elsewhere [40]. The software personalizes the progression of the exercises according to the individual’s physical capacity. It establishes the volume of training, the criteria for progression, and the guidelines for interrupting the protocol, thus resembling a face-to-face intervention. A total of 104 exercise variations comprised of 1) muscle stretching, 2) strengthening of the intrinsic muscles of the foot, 3) strengthening of the extrinsic muscles of the foot and ankle, and 4) functional exercises (e.g., balance and gait training) are available through the software (Fig. 3).

The progression of the exercises is based on individual capabilities, which are graded by a visual analogue effort scale after each exercise is performed (Fig. 3). The discontinuation criteria for the exercises include cramps, moderate to intense pain, fatigue, or any other condition that exposes the patients to any discomfort.

**Fig. 4:**
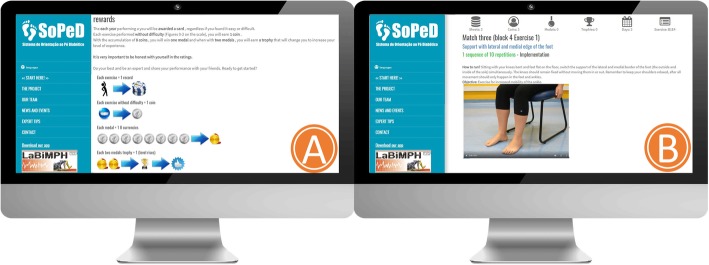
Diabetic Foot Guidance System (SOPeD) interface. (**a**) Game rules. (**b**) Exercise page in game format

To improve adherence, the SOPeD was developed using gamification components (Fig. 4) to encourage patients to keep using the tool [59].

**Fig. 5:**
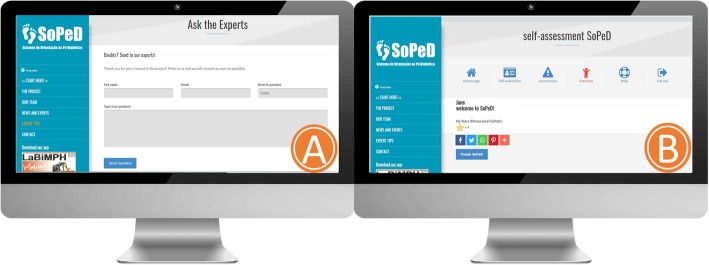
Diabetic Foot Guidance System (SOPeD) interface. (**a**) Section for communication with specialists. (**b**) Communication forum

Users also have the opportunity to send messages to the main researcher or any member of the specialized team about their training, DPN, or any technical difficulties while using the software. The user can also participate in a forum to exchange experiences with other people who are in the same group as them (Fig. 5).

One discontinuation criterion for the intervention is the occurrence of a foot ulcer as assessed by a blinded podiatrist nurse who specializes in diabetic feet. The participants will be advised to report to Researcher 3 with any sign of tissue damage.

If an intervention group participant fails to log in to the web-based software for more than 3 consecutive days, an email will be automatically sent asking the participant to log in to their account. The physiotherapist responsible for the therapeutic protocol will also make telephone contact with participants who do not respond to email reminders from the web-based software. If an intervention group participant fails to access the software for 2 consecutive weeks without explanation, that participant will be terminated from the study.

After the 12-week intervention period and follow-up, all participants will be questioned about their satisfaction with the protocol: “Did you enjoy doing the exercises?” There will be three possible answers: no, a little, and a lot. To avoid evaluation bias, participants will answer this question secretly through an anonymous online form sent to their email. Patients will be informed about their anonymity, and this form will only be accessed after the completion of the study.

For the duration of the trial, participants from the intervention group and control group will be advised not to engage in any new physical therapy program for the foot and ankle. If any participant cannot avoid such behavior, they must report this situation to the main researcher and will be excluded from the study.

### Data management

The study steering committee is comprised of two PhD students (blind evaluators), two master’s students (responsible for data collection), two undergraduate students (responsible for data tabulation and codification), a coordinator (responsible for managing the project) and an assistant Research (responsible for the recruitment and scheduling of collections).

All information collected during the protocol will be entered onto an electronic form by those responsible for data collection. The integrity and validity of the data will be verified at the time of data entry (edit checks). Identification of potential recruits will be done by the project manager and the research assistant. The research assistant will be trained on how to approach the eligible participants during the initial recruitment contact for the survey (made by telephone calls) and how and when to contact them for follow-up and data collection.

### Oversight and monitoring

The Data Monitoring Committee (Steering Committee) and the Faculdade de Medicina da Universidade de Sao Paulo Board will regularly monitor (depending on the recruitment numbers and collections performed) the study datasets and make recommendations on necessary protocol modifications or termination of all or part of the study. A trimester meeting is held to facilitate the study development. All team members can request meetings as needed.

All adverse events occurring during the clinical trial period will be recorded. Minor adverse effects potentially expected are muscle soreness and tiredness after performing the proposed exercises. The patients will be advised to report any discomfort and foot preulcerative signs (blisters, callus, or even foot ulcers) to Researcher 3 who will ask for the blinded podiatrist nurse to assist the patient.

### Sample size and statistical analyses

The sample calculation was performed using the GPower v.3.1 program [60]. Two outcomes of extreme functional importance for patients with DPN were used to calculate the sample size. Considering the primary outcome (DPN symptoms), a medium effect size (0.52) was adopted and, for the secondary outcome (peak pressure at forefoot), a small effect size (0.20) was adopted. Both effect sizes were taken from the study by Sartor et al. [30] which evaluated the effect of a 12-week supervised physical therapy exercise in patients with DPN. In order to obtain the largest sample size, the smallest effect size (0.20) was used. A statistical design of F-test repeated measures and interaction between and within factors with two repeated measures and two study groups, a statistical power of 0.80, an alpha of 0.05, and an effect size of 0.20 were used for the sample size calculation. The resulting sample size was 52 individuals. A final sample size of 62 patients was then chosen after estimating a drop-out rate of 20%.

The inferential statistical analysis will be based on an intention-to-treat analysis and per-protocol analysis. Mixed general linear models of analysis of variance for repeated measure will be used to detect treatment–time interactions (α = 5%), and the Newman–Keuls post hoc test will be used to obtain group effect (intervention group and control group), time effect (between T0 and T12), and group–time interaction. Effect sizes (Cohen’s *d* coefficient) will also be provided between T0 and T12 and between T12 and T24 to determine if the intervention shows any treatment effect. The difference between the means with their respective 95% confidence intervals will also be calculated. Imputation of any missing data for the analyzed variables will be conducted depending on the nature of the losses: missing completely at random, missing at random, or missing not at random. The per-protocol analysis will include only those patients who completed follow-up in the allocated intervention group. If there is evidence that the difference in the treatment depends on certain patient characteristics identified in the baseline assessment, a subgroup analysis will be performed.

## Discussion

This paper describes the FOCA trial I protocol which will test the customized SOPeD with the aim of reducing DPN symptoms and classifying DPN severity and gait biomechanics. As there is no evidence about the efficacy of software focusing on foot-related musculoskeletal deficits in reducing DPN symptoms and DPN severity or for promoting benefits in biomechanical, clinical, and functional outcomes of people with DM and DPN, this research can contribute substantially to clarifying the therapeutic merits of rehabilitation technology interventions.

The existing clinical trials that have proposed specific exercise protocols to minimize musculoskeletal disorders resulting from DPN have shown promising results in reducing DPN symptoms, promoting more physiological plantar pressure distribution, and increasing muscle strength and joint mobility, but these benefits were not sustained at follow-up [29, 30, 33, 34, 61]. Our hypothesis is that this is due to the interruption of the exercises; this is consistent with the principle of reversibility of physical training, which states that discontinuation of training causes a decrease in physical abilities [62]. An alternative is protocols that can potentially improve compliance and encourage participants to exercise even when not directly supervised by a physical therapist.

Another alternative to increase adherence and stimulate lasting self-management is the insertion of health technologies in the patient’s routine. A scoping review evaluated 47 articles that showed that the use of technologies has positive impacts on the self-management processes of people with DM, such as adherence to blood glucose monitoring, day-to-day decision-making related to self-care, and adherence to medications [63]. This practice is a worldwide trend and widely encouraged by the American Diabetes Association, which recommends the use of technology-assisted therapies, including the Internet, distance learning, and mobile applications. This recommendation is made because technology-assisted therapies are useful strategies for modifying the lifestyles of people with DM as well as increasing their adherence to the proposed treatments [39].

The fact that the proposed treatment will be performed without the supervision of a physical therapist could be considered a limitation. However, it is very important for DPN patients to develop independence and autonomy to stimulate self-care attitudes, and the SOPeD has the potential to achieve this goal [40].

The exercise protocol contained in SOPeD is fully focused on musculoskeletal deficits resulting from DPN and is based on decades of biomechanical research in this population [10, 11, 14, 46, 64–66]. Thus, we expect to contribute to minimizing the deleterious consequences of DPN on patients’ daily living activities with the use of SOPeD as a self-care strategy. If this easy-to-use tool and therapeutic exercise protocol proves effective for reducing or attenuating the musculoskeletal and sensorial deficits caused by DPN, it could be easily incorporated into patients’ usual daily care routines.

This rehabilitation technology is now available free and is intended to be implemented in public health settings after its effectiveness has been demonstrated. SOPeD could fill an important treatment gap in the provision of health services to a large group in need, thus significantly improving care for a large population with DPN. In addition, after implementation in public health in Brazil, the tool may prove useful for people with DM and DPN all over the world. It is currently available in Portuguese and English and can be translated into any other language.

## Trial status

ClinicalTrials.gov identifier NCT04011267, version 1.0, July 8 2019. Registered on 8 July 2019 and last updated on 9 September 2019. Participant recruitment began on 1 August 2019 and is expected to continue until the middle of 2021. Randomization of the participants was performed on the same day.

## Supplementary information

**Additional file 1:** SPIRIT 2013 Checklist: Recommended items to address in a clinical trial protocol and related documents*.

## Data Availability

All personal data from potential or enrolled participants will be maintained confidentially before, during and after the trial by encoding the participant’s name. All data access and storage are in keeping with National Health and Medical Research Council guidelines, as approved. All files will be available from the database published at figshare.com. The main researcher will report all important protocol amendments to investigators, review boards and trial registration. Upon the completion of study, supported data will be available upon request.

## Abbreviations

CONSORT: Consolidated Standards of Reporting Trials
DM: Diabetes mellitus
DPN: Diabetic peripheral neuropathy
FHSQ-BR: Brazilian version of the Foot-Health Status Questionnaire
OFM: Oxford Foot Model
SOPeD: Diabetic Foot Guidance System
